# Emotional Availability in Mother-Child and Father-Child Interactions as Predictors of Child’s Attachment Representations in Adoptive Families

**DOI:** 10.3390/ijerph19084720

**Published:** 2022-04-13

**Authors:** Ana Susana Almeida, Jean-Christophe Giger, Sandra Mendonça, Marina Fuertes, Cristina Nunes

**Affiliations:** 1 Psychology Research Centre (CIP) & Universidade do Algarve, Campus de Gambelas, 8005-139 Faro, Portugal; jhgiger@ualg.pt (J.-C.G.); csnunes@ualg.pt (C.N.); 2 CDI.UP—Cooperativa de Desenvolvimento Infantil e União Parental, CRL, 8125-196 Quarteira, Portugal; svm.mendonca@gmail.com; 3 Centro de Psicologia da Universidade do Porto (CPUP) & Escola Superior de Educação (ESELX-IPL), Campus de Benfica do IPL, 1549-003 Lisboa, Portugal; marinaf@eselx.ipl.pt

**Keywords:** adoption, adult-child interactions, emotional availability, secure attachment representations

## Abstract

Emotional availability (EA) in parent-child interactions is associated with positive child outcomes, including attachment security. However, little is known about EA in adoptive families. This study investigated the associations between secure representations of attachment in adopted children and the adoptive parents’ EA. The participants (*n* = 75) included 26 mothers, 23 fathers, and 26 children who were aged 3 to 9 years. Children completed the Attachment Story Completion Task. Adult-child dyadic relationships were assessed using the EA^®^ System. The results showed that the children’s and parents’ EA, age when adopted, and time elapsed since adoption were associated with more secure children’s attachment representations. Implications for family support and public policy are discussed.

## 1. Emotional Availability

Interactions between mother and child have been compared to an elaborate dance between two partners [1]. The emotional quality of this interaction has been shown to have a profound effect on the child’s development [2,3]. Indeed, the ability to manifest emotional availability to the interactive partner is essential to the quality of the dyadic interaction [4,5].

**Figure 1:**
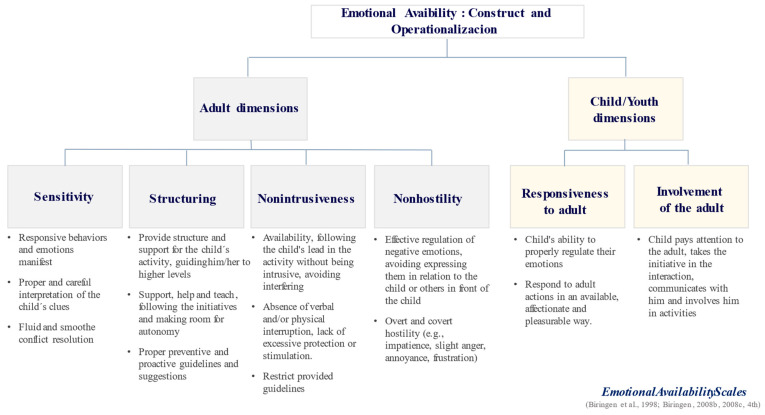
Dimensions of emotional availability (EA).

Emotional availability (EA) is defined as a dyadic and relational construct, which refers to both the emotional quality of the relationship and the ability of each element of the dyad to affect the other element [6] in a systematic exchange of interactions loaded with communicative messages—verbal and non-verbal, active or passive, consistent or inconsistent—that create an interactive spiral. EA consists of the ability for dyadic partners to experience healthy emotional connections [7]. This construct is multidimensional [7]. It integrates four adult dimensions (i.e., sensitivity, structuring, non-intrusiveness, and non-hostility) and two child dimensions (i.e., responsiveness and involvement) [8,9] (see Figure 1). EA encompasses a wide range of specific aspects that are equivalent in terms of value, complexity, and predictive value [8]. Indeed, EA includes not only attachment ingredients, such as responsiveness and sensitivity, but also other important relational aspects that have been shown to be associated with the quality of everyday interactions (e.g., regulation of negative emotions, existence of an emotionally positive environment, preventive limits setting, promotion of healthy autonomy, and support of the child’s interests) [9].

EA contemplates the quality of both adult-to-child and child-to-adult interaction behaviors, since they can match or mismatch [10]. For example, the adult can display positive interactive behaviors toward the child, and the child can display negative interactive behaviors in response. Moreover, it is assumed that both actors influence one another in a bidirectional manner. Therefore, the quality of the connection lies not only in the adult’s immediate actions but also in the child’s response [11]. Accordingly, consideration of the behaviors observed in both adult-to-child and child-to-adult interactions can better reflect the reality of the interaction, and the emotional and dyadic quality of the relationships [10]. Interestingly, the emotional exchanges between caregivers and their child shapes the attachment relationship [12], which makes these two concepts closely associated.

### 1.1. Emotional Availability and Attachment

Biringen’s EA conceptualization is closely associated with attachment. It constitutes an extension of the initial conceptualization of the child’s primary attachment relationships [10], and it reflects the emotional and health aspects that underly secure attachment (EA) [8,13,14,15,16].

Several investigations have highlighted the significant relationships between EA and attachment among participants of different nationalities, ages, and psychosocial risk status [6]. EA has been shown to be either a correlate of attachment or a predictor [6,17].

Children whose mothers showed greater sensitivity in interacting with them had more secure attachments and, conversely, children whose mothers exhibited less sensitivity tended to establish an insecure attachment with their caregivers (see [18,19,20,21,22,23,24,25,26,27,28,29]).

Ziv et al. [29] highlighted the relationships between different dimensions of EA (i.e., sensitivity, structuring, non-intrusiveness, responsiveness, and involvement) and attachment security. Meanwhile, Swanson et al. [30] revealed that intrusiveness exhibited by mothers who abused substances was a predictor of insecure attachment in their children at 18 months.

Some studies have also revealed the relationship between EA and disorganized attachment [6]. Swanson et al. [30] used the strange situation procedure during 10 min of free play interaction and found a negative relationship between maternal non-intrusiveness and disorganized attachment of the respective children. Similarly, a longitudinal study by Easterbrooks et al. [31] showed a relationship between EA and a child’s insecure attachment measured at 12–18 months and 7–8 years old. Other studies have revealed the differential associations between EA and maternal attachment styles (see [32]).

Ziv et al. [33] evaluated the impact of a program based on the attachment construct to improve the dyadic EA of mothers and children (aged 3–6 years). They observed significant differences between pre- and post-intervention in maternal sensitivity and structuring, as well as in the responsiveness and involvement of children in relation to their mothers. These results highlight the important association between these two constructs. This association is of particular importance in adoptive families, where new emotional relationships and interactions will emerge [34].

### 1.2. Attachment in Adoptive Families

Children who integrate their new families by adoption can bring with them a large amount of “*baggage*”, and a history that is characterized by various degrees of adversity and experiences of “*separation and loss*” (e.g., abandonment, abuse, neglect, and institutionalization), even when adopted at an early age ([35], p. 362; [36]). For example, children who are adopted at birth can experience some degree of adversity, such as in utero exposure to stress (e.g., [37]) or postnatal separation from the biological mother that imprints the infant with a sense of abandonment and loss [36]. Siegel [38] suggests that an adopted infant’s brain synapses begin connecting according to a perception of the environment (e.g., unsafe, scary, and in need of vigilance). For children who are adopted later in life, the experience of institutionalization that should be a safeguard and security protective measure for children can be an experience with harmful and compromising effects on the construction of a secure relationship [39].

A vast body of research has consistently indicated that children exposed to neglect and trauma at an early age tend to form negative representations of others and of themselves [40,41,42]. These previous experiences contribute to forming their internal working models [34]. These models constitute the internalized relational pattern that will guide the way in which adopted children identify, interpret, and behave with themselves and with others, and may influence the relationships that they establish with the adoptive family [34]. Children who build their internal working models through experience with a caregiver who exhibits a lack of responsiveness and interest in their needs will tend to focus on clues that confirm their internal representations to interpret new experiences [41,42].

When used systematically, the internal working models achieve stabilization and progressively become autonomous, gradually activated in non-conscious ways [43], which make them difficult to update [34,44,45]. Two aspects are related to the adopted child’s life course that are associated with differences in the updating of the internal representations of adopted children—the age and time of adoption [41,42].

Within the scope of adoption, one of the most explored risk factors in research is the child´s age when adopted, which is assumed to be a relevant predictor of adoption outcomes [40]. For example, early adoption is an important childhood protector from the risk of adulthood depression (which is associated with early adversity) [46]. A diverse set of research findings indicate that adoption in the first few months of a child’s life is associated with establishing secure attachment relationships (e.g., [47]). Conversely, adoption at later ages appears to increase the risk of building insecure attachment relationships (e.g., [41,42,48,49]) and tends to be associated with a diverse range of problems in children (e.g., behavioral, socio-emotional and adjustment) [50]. Hodges et al.’s [41] longitudinal research suggests that attachment representation is even more stable (difficult to change) in children who are adopted between six and eight years of age (compared to children adopted between four and six years of age). Conversely, children adopted before the first year of life consistently showed more positive representations and fewer negative representations (compared to children adopted after the first year of life). However, some studies have emphasized the lack of association between the child’s age when adopted and the security of attachment with the adoptive parents, which was assessed using the child’s attachment representations (e.g., [51,52,53,54,55,56,57]).

It is possible that the influence of the adoption age is not directly associated with the attachment representations, but is rather influenced by the impact of the time needed to consolidate the bond in the post-adoption period [58]. Reconstruction of security in relationships will be faster at the behavioral level compared to the representational level [34]. In fact, many empirical findings suggest that the amount of time that the child remains in the adoptive family is associated with the attachment security [41,42,49,59,60,61,62]. For example, Hodges et al. [41] and Steele et al. [42], in a longitudinal study, asked adopted children (*M* = 6 years, ranged from 4 to 8 years, with a history of abuse or severe neglect) to solve dilemma stories about a hypothetical family in daily family scenarios. Across two years, they found that although children were still presenting their initial negative representations about their family (stemming from their past), they also displayed progressive positive changes on some aspects related to expectations and perceptions of family rules, attachments, and relationships. The authors suggest that these changes probably reflect positive caregiving by the adoptive parents. In brief, changes towards more positive representations are not immediate, but are slow and gradual, and need time.

The cumulative history of emotionally significant interactions may enable the reconstruction of previously internalized attachment models [63]. However, the interactive parental behaviors that give quality to the attachment and which allow the construction of a secure bond with the child are still poorly understood. Therefore, it is important to investigate attachment representations and parental interactions with children [57].

### 1.3. Emotional Availability and Attachment in Adoptive Families

Research has reported the relationship between EA, operationalized with emotional availability scales (EAS), and attachment in adoptive families. Overall, the results are mixed. For example, Altenhofen et al. [64] found that the mother’s sensitivity, and the child’s responsiveness and involvement (at three years old) were related to the child’s secure attachment, measured with AQS [65]. The authors were able to explain 16% of the variance related to secure attachment when the child’s gender was controlled. However, the results were not exclusively for adoptive families, since families with very heterogeneous characteristics participated in the study (i.e., 14% were biological parents, 23% were other biological relatives, 49% were adoptive parents, and 14% were foster care families). Piermattei et al.’s [55] study included 20 adoptive families, with children aged between 4.5 and 8.5 years old, and considered both mother-child and father-child dyads. Their results showed that insecure mother-child dyads (assessed with a Manchester Child Attachment Story Task for children) displayed less emotional availability (measured with EAS) than the secure dyads. Additionally, Baker et al. [66] evaluated the effectiveness of the new tele-intervention program “*Emotional Attachment and Emotional Availability (EA2) Intervention*” [13,14] in the integration phase of the adopted children aged between 1.5 and 5 years, with a post-adoptive service. The results after 6 weeks of intervention comprising five sessions of about 45 min of video viewing with relevant information about EA and attachment, in addition to times of joint discussion between families, indicated the following: (1) the responsiveness and involvement in relation to adoptive parents (dimensions of adopted children) increased significantly and substantially; (2) there were no significant differences in parent-reported child attachment safety behaviors (Attachment Q-sort, version 3.0; AQS; [67]), although a progressive increase in mean safety outcomes was observed, with moderate levels; (3) there was a significant increase in the observed emotional attachment of children (filming coding through the Emotional Attachment & Emotional Availability Clinical Screener—EA2-CS; [8]). Inversely, the results of Van Den Dries et al.’s [68] study with adoptive families (children aged 15 to 19 months) indicated the absence of strong relationships between the attachment of adopted children (strange situation—SSP; [2]) and the sensitivity of the adoptive mother/father. Other studies investigated EA in adoptive families (see [69,70]); however, attachment was not considered and thus these studies are not contemplated here.

Interestingly, studies in adoptive families have mainly focused on mother-child dyads and rarely on the father-child dyad (see for exceptions [55]). Studies of the father-child dyad in non-adoptive families are also scarce (see for exceptions [71,72]). Accordingly, little is known about the relationship between EA and attachment within the father-child dyadic interaction.

In summary, the link between EA and attachment representation is still understudied in adoptive families. New investigations that focus on the relationship between EA in adoptive families and the attachment of children are needed. It is important to consider both mother-child and father-child dyads, in a wide range of adoptive child´s ages.

### 1.4. Current Study

This study is part of a larger research project “*Adjustment in Adoptive Families*” whose objective is to describe, using a quantitative and qualitative approach, the individual and family aspects that best contribute to greater EA, a more positive parental perception of their experience, and a better individual and family adjustment in adoption. Currently, there are only a handful of studies that explore the relationship between EA and children’s attachment in adoptive families (see [55,63,66,68]). Recent research has highlighted the advantages of using narratives to access the children´s internal working models [40]. However, only a few studies have explored the EA in adoptive families and the relationship between dyadic EA, and the attachment representations in both mother-child and father-child interactions. Accordingly, this study aims to investigate the association between EA in interactive adoptive dyads (i.e., mother-child and father-child) and secure attachment representations of adoptive children, considering the age of the child, the age of the child when adopted, time with the adoptive parents, and the parents’ gender.

## 2. Participants, Materials, and Procedures

### 2.1. Participants

The total sample (*n* = 75 Portuguese participants) consisted of 26 children (*M*_age_ = 74.77 months; *SD* = 19.61), including 7 girls (*M*_age_ = 60.00 months; *SD* = 14.92) and 19 boys (*M*_age_ = 80.21 months; *SD* = 18.54), in addition to 49 parents (*M*_age_ = 40.04 years, *SD* = 3.69), including 26 mothers (*M*_age_ = 39.33 years, *SD* = 3.60) and 23 fathers (*M_a_*_ge_ = 40.74 years; *SD* = 3.72). Among the children, four were 3 years old, two were 4 years old, four were 5 years old, seven were 6 years old, five were 7 years old, three were 8 years old and one was 9 years old. In total, 23 children were previously institutionalized (the previous situations of three children were unknown since the parents decided to not report them). The information related to adoption (e.g., age when adopted) is displayed in Table 1. In total, 23 families were biparental (one female homosexual family) and three were single parent families. Moreover, 21 families had no biological children, while five did. Two biological children were female (*M*_age_ = 114.00 months; *SD* = 93.33), and three were male (*M*_age_ = 95.00 months; *SD* = 54.69).

This study was part of a larger funded project that aims to determine the relationships with EA, and the child’s and parents’ characteristics. As a result of the inclusion criteria of one of the tasks (i.e., the Attachment Story Completion Task; [73]), only children between 3 and 9 years old, and their respective parents, were included in the current study. Data collection took place before the COVID-19 outbreak.

### 2.2. Measures

*Emotional Availability (EA).* EA is assessed by the EAS (see [8,14,15], 4th edition). Since its elaboration, EAS has been widely used; see [6,9,10] for a review. This is one of the most commonly used instruments for assessing the interactive quality of the parent-child dyad [9]. EAS is an observational system [9] that assesses the adult’s (i.e., caregiver´s) and child’s EA, their behaviors, and affections [7]. This is a set of important aspects of the relationships that is displayed at a particular moment in time [9]. EAS is a method that aims to characterize the emotional quality of parenting through the observation of adult-child dyadic interactions. EAS can used in very different age groups (e.g., from pregnancy to 14 years) [9]. Interaction was coded according to a multidimensional framework [7] that integrates six scales assessing each of the six dimensions related with emotional regulation in the adult-child dyad; see [14,15], 4th edition. Four scales are used to assess the EA of the adult in relation to the child: sensitivity, structuring, non-intrusiveness, and non-hostility, and two assess the EA of the child in relation to the adult, namely, responsiveness and involvement [9]. The EAS has been widely used to assess EA through observations and has been proven to be a reliable instrument (see [6,9,74]). All EAS scales displayed a good internal consistency (see Table 2).

*Secure attachment representations.* Infant attachment representations were evaluated with the Portuguese version [75] of the Attachment Story Completion Task (ASCT; [73]). The ASCT is a semi-projective methodology for 3- to 6-year-old children [76], but can be applied for ages up to 9 years with slight adaptations (see [77]). This is one of the most commonly used narrative methodologies during the preschool period and during the transition to school [78]. The ASCT displays a good agreement with other early childhood measures that assess the safety of the child’s attachment behavior to the mother (e.g., [73,79,80,81,82]).

The ASCT is based on the completion of six stories that are re-enactments of everyday parent-child interactions. One story is used for training and five are target stories. Each story presents a central problem that aims to activate the child’s attachment representations. The procedure for school-aged children included some changes in the histories (e.g., a monster was changed to a frightening figure in the “*monster history*”; “*I’m bleeding*” was added on the “*hurt knee history*” and the separation period was increased to 3 days; older brothers were removed in the “separation history”) to allow the activation of feelings related to attachment in older children [75]. The average time of application of the stories is about 20 to 30 min. The total security of the child’s representations was calculated on the mean of the scores obtained in each story [75,78]. In the present investigation, the data obtained through this procedure showed good internal consistency (see Table 1).

*Socio-demographic questionnaire.* As part of a larger survey, the parents were asked to provide socio-demographic information. Data about parents and their adopted child; gender and age of each member of the family; child age when adopted; the time elapsed since adoption, as well as since previous institutionalization were collected (see Table 2).

### 2.3. Procedures

#### 2.3.1. Participant Selection and Contact

The investigators contacted and presented the study to the team “*Adoção, Acolhimento Familiar e Apadrinhamento Civil*” of the Social Services of the Algarve region (south Portugal). This team directly contacted and presented the objectives and the methodology of the study to all adoptive families of the south region. Most of the contacted families (80%) accepted to participate in the study, and the social services departments then transmitted their contact details to the investigators. The investigators contacted the families, presented again the objective and methodology of the study to them, sent them a questionnaire, and these families were asked to sign the informed consent form. A visit at home was then arranged. During the visit (about 5 h), the investigators first collected the already filled tests, and then videotaped the adult-child dyadic interactions and the Child Attachment Story Completion Task.

#### 2.3.2. Video Recording and Coding of Data

*Emotional availability*. Parents were asked to choose a place in which they usually interact with their child. A camera with a tripod was installed, and the parents were asked to play and interact with their child normally. The mother-child and father-child dyadic interactions were videotaped separately. Toys were provided.

Two trained raters coded the entire adult-child free play of all dyadic interactions according to the fourth version of the EAS (EA^®^ System, https://emotionalavailability.com/ [8,14,15], accessed on 25 March 2022). Authors 1 and 4 had previously received a certificate to use and code EA sub-dimensions directly from Zeynep Biringen (i.e., *EA^®^ Distance Training and Certification*). This training included the achievement of reliability with Zeynep Biringen. The indirect method of coding was used to assess EA dimensions, in which each dimension is assessed by 7 or 8 features. The sum of the scores on each feature provides the total score of each of the six dimensions (see [8,14,15]). Moreover, 10% of videotaped interactions were randomly selected in order to calculate inter-observer agreement (see [6] for a similar procedure). The results showed a 97.17% agreement with an overall intraclass correlation coefficient mean of 0.80.

*Children’s symbolic representations of attachment.* In the ASCT [73], children were asked to complete six stories. The investigator began each story, and the child had to continue and finish it using a family of dolls and other materials (e.g., small furniture, a car, and other miniature objects; see [79]). Children were videotaped while completing the stories. Two observers with Bachelor’s and Master’s degrees in psychology were previously trained to apply and code the ASCT. Both watched, separately, the filming with the stories of each child, and used the instructions available in the manual in Portuguese [75] to score the adopted children’s stories. Each child´s narrative is coded into three specific parameters: (1) the resolutions of the story (i.e., the quality of the resolution, where “1” refers to a “*minimal resolution*”, “2” to a “*full resolution*”, “3” is equivalent to an “*unresolved story*” and, finally, “4” is assigned to a “*turnaround resolution*”); (2) the consistency of the story (rated from “1” “*extremely inconsistent*” to “8” “*very coherent*”); and (3) the total security (rated from “1” “*disorganized*” to “8” “*very safe*”) [76,79]. The total security of the children’s representations was calculated using the average of the scores obtained from each story [75]. Inter-observer agreement was calculated on 20% of the collected data. The results showed an average agreement of 92.5%, with differences of one value ranging between 87.5% and 100%. This is indicative of a very acceptable degree of reliability. The weighted kappa coefficient ranged between 0.38 (mild) and 0.88 (good), with an overall average of 0.58 (adequate), with the lowest values being related to the stories “injured knee” and “monster in the bedroom”.

## 3. Results

### 3.1. Preliminary Analyses

Table 1 displays the descriptive statistics of the variables under study. All of the variables showed good indicators of skewness and kurtosis (i.e., below +1.5 and above −1.5; [83]). A series of Mann–Whitney tests were conducted in order to test the gender differences on the dimensions of EA. The results revealed that only child involvement was higher in mother-child dyadic interaction (*Mdn* = 29.08) than in father-child dyadic interaction (*Mdn* = 20.39), *U* (26;23) = 193.00; z = −2.13, *p* = 0.033.

Table 2 shows the children’s characteristics. All of the variables displayed acceptable indicators of skewness and kurtosis. A Mann–Whitney test indicated that there were no differences in the secure attachment representation index between adopted girls (*Mdn* = 13.14) and boys (*Mdn* = 13.63), *U* (7;19) = 64.00; z = −0.14, *p* = 0.91.

### 3.2. Association between Emotional Availability and Children’s Secure Representations of Attachment

#### 3.2.1. Correlational Analyses

Table 3 displays correlations between secure representations of attachment and EA, separately for mothers (upper right-hand corner) and fathers (lower left-hand corner). In the group of mothers, children’s secure representations of attachment were positively correlated with involvement, responsiveness, and their synthetic index (i.e., EA-child). In the group of fathers, children’s secure representations of attachment were only positively correlated with involvement.

#### 3.2.2. Regression Analyses

The predictive power of the EA-parents and EA-child on adopted children’s secure representations of attachment was tested with a series of independent linear regression models, with EA-parents and EA-child as main predictors and two control variables. This decision was taken as a result of the small sample size (*n* = 49), the criteria of 10–20 observations per estimated parameter (covariate) (see [84]), and problems of multicollinearity between the EA subscales (see [64] for a similar problem with EAS). Moreover, the sample size did not allow us to conduct more complex statistical procedures, such as structural equation modeling (SEM), which would allow us to test relationships between all variables at the same time.

Previous studies have identified gender, child’s age, child’s age when adopted (see [41,42,47,48,49,62]), and the time elapsed since adoption (see [41,42,56,59,60,61,62]) as important factors in adoption adjustment. Consequently, they were considered as control variables in the different models. The gender of the children was not considered since the result of preliminary analyzes showed a lack of difference between boys and girls regarding secure attachment representations.

Table 4 presents the results of regression analyses. No multicollinearity problem was observed (all indicators with tolerance > 0.10 and VIF < 10). Globally, EA-parent was a positive and significant predictor of secure attachment representations, even when child age when adopted, child age, and time elapsed since adoption were controlled (see models 1 and 2). Moreover, EA-child was a positive and significant predictor of secure attachment representations, even when age when adopted, parents’ gender, and time elapsed since adoption were controlled (see models 3 and 4). In short, the better the EA, the more secure the child’s representations of attachment.

## 4. Discussion

This study examined the association between EA and a child´s secure attachment representations in adoptive families. The dyadic and bidirectional interactions between adoptive parents and adopted children (i.e., mother-child and father-child) were considered. The age of the child, age of the child when adopted, time with adoptive parents, as well as parents’ gender were controlled. The multivariate results revealed the following:

(a) EA-parent (i.e., sensitivity, structure, non-intrusiveness, and non-hostility) was a significant predictor of the child´s secure attachment representations, even when child age when adopted, child age, and time elapsed since adoption were controlled (see models 1 and 2). These factors, taken together, explain 49% of the variance (see models 1 and 2) in the security of the adopted child attachment representations.

(b) EA-child (i.e., involvement and responsiveness toward the mother or father) was a significant predictor of the child´s secure attachment representations, even when child age when adopted, child age, and time elapsed since adoption were controlled. Taken together, these variables explain 19% and 42% of the variance (see models 3 and 4, respectively) in the security of the adopted child attachment representations.

These results support the claim that the emotional interactive experience underpins secure attachment relationships. They also highlight the importance of EA of new attachment figures in interactions with their child. Additionally, the results confirm that children with discontinuous trajectories (e.g., from biological family, institutionalization, and to adoptive family) can construct secure representations of attachment.

In the child-mother dyad, the results show that the total EA-child, and involvement and responsiveness, individually considered, were positively correlated with secure attachment representations. More specifically, while interacting with their mothers, adopted children who exhibited (a) greater responsiveness (i.e., clear display of pleasurable interaction, enthusiasm in the interaction with the adoptive mother, behavior of organized affection, well-regulated emotions, and the search for opportunities to be exploratory and autonomous), and (b) greater involvement (i.e., involving the mother in their activities, taking the initiative to bring her into the interaction, making the mother their audience, sharing interests, and positioning the body towards the mother), were also those who tended to:

(1) Reveal more complex narratives, with a relational and emotional understanding of the problem;

(2) Find adequate, easy, coherent, constructive, and imaginative solutions to solve problems, and include a resumption of interaction to normality and harmony [75].

These results echo those found in North America by Altenhofen et al. [64], whose participants were, among others, adoptive families. The authors showed that children´s responsiveness and involvement in dyadic interactions with mothers were associated with secure attachment (measured with AQS; [65]).

Interestingly, in the child-father dyad, only child involvement was positively correlated with secure attachment representations. These findings differ from other research in which no significant associations were found for father-child relationships and child attachment patterns (e.g., [85,86]).

Moreover, our results show that there was a difference in EA quality as a function of parental gender: child involvement was higher in mother-child dyadic interaction than in father-child dyadic interaction. This result diverges from Bentenuto et al. [71], who found no differences in EA between fathers and mothers, and Ellis-Davies et al. [72], who concluded that mothers’ and fathers’ EA sensitivity and intrusiveness were similar. However, our results are compatible with Brown et al. [87], who assumed that the father-child and mother-child relationships can be centered on different aspects.

The difference in EA quality in different dyads (child-father and child-mother) is also evidenced in the fact that this study’s results show that involvement of the child was of higher quality in the child-mother dyads compared to the child-father dyad. During the home visits that were carried out to collect data for this study, the adoptive families shared several stories about their adoptive processes. Among them, several families reported the differential difficulty in establishing the relationship between mother and child, and father and child. They reported a much greater difficulty for adoptive fathers when establishing a relationship with their children, due to greater rejection from the child, and even anxiety and fear that the child showed for several months after adoption in relation to the adoptive father. Some of these families suggested that the child had regular and long-lasting interactions with female adults in their temporary shelter institution. In some of these institutions, the entry and/or stay of male adults was not allowed. Accordingly, parents reported that the child found that it was “strange” to interact with male individuals, which made it very difficult to establish a relationship with the adoptive father. This aspect was mentioned by several families and should be considered since it could contribute to understanding the greater quality of the child’s involvement in their interactions with the mother compared to their interactions with the father.

The results of the present study regarding differences in involvement echo those found in the study by Lovas [88] with non-adoptive families involving children aged 18–20 months. Thus, it is important for future investigations to explore this difference in involvement exhibited by the child in relation to the father and mother. However, this trend is not always observed. For example, in the study by Bentenuto et al. [71], children (*M* = 41.6 months with autism spectrum disorder) showed similar involvement and responsiveness results for both interactive dyads (i.e., mother-child and father-child). Finally, the multivariate results of the present study (see models 1 and 2) reiterate the need to consider aspects, such as the child´s age of adoption and time in the adoptive family, when investigating the ingredients associated with the internal representations of adopted children (see also [41,42,62]).

### 4.1. Limitations

Although the present findings contribute to the understanding of adoptive families’ relational functioning and the child’s adjustment, some methodological limitations should be considered. The sample is geographically restricted to the south of Portugal and, accordingly, the generalization of the results should be made with caution. Although the sample included 80% of the total available adoptive families in the region of Algarve, its small size did not allow the use of sophisticated statistical tests (e.g., SEM) that would allow us to simultaneously explore the relationships between all the variables. The low variability in family composition (e.g., presence/absence of biological children and other adopted children) did not allow us to explore aspects that may also influence dyadic parents-child EA and the child’s attachment. Moreover, because of the quick development of cognitive and affective capacities between 3 and 9 years old, further studies should explore the association between EA and attachment in specific age subgroups (e.g., 3–4, 5–6, or 7–9 year-old children). The parents’ individual characteristics (e.g., mental health, parents’ educational memories, history of early life maltreatment; see [89,90]), parental relationship quality, as well as children’s individual characteristics (see [71]) may influence the parents’/children´s EA and/or its relationship with child attachment, and should accordingly be considered in future studies. More distal aspects (e.g., socio-cultural and contextual factors) also need to be considered when investigating parenting (see [91]). Finally, the present study is cross-sectional; further studies should use a longitudinal design in order to explore the evolutive trajectories in attachment (e.g., attachment at the time of placement).

### 4.2. Practical Implications

The relevance of EA on the adopted child secure attachment representations is corroborated by Wiefel et al. [92], who argued that the operationalization of EA is a useful tool for better understanding the functioning of the child, the family, and the relationship established between them. Its practical relevance is highlighted in the intervention setting (e.g., diagnosis process, decision-making about the most suitable interventions for each case, and feedback in sessions with families). In fact, EA conceptualization and operationalization (by the Biringen team) is useful for preventive intervention and treatment-focused interventions; EA conceptualization is currently widely used in diverse contexts, such as by therapists (e.g., evaluating the therapeutic process) and other professionals working with families (e.g., social service and child custody situations) [9].

Our results highlight the relevance of intervening with adoptive families to promote better parental EA (i.e., sensitivity, structure, non-intrusiveness, and non-hostility) and better EA for the children. It is important that parents are sensitized to the need to identify, promote, and reinforce the responsiveness and involvement exhibited by children towards them. Adoptive parents can promote their child’s skills of self-regulation of emotions, modeling of empathic behaviors [93], instigating opportunities for the child to have exploratory and autonomous behaviors, and encouraging the active sharing of interests and pleasure in joint activity, whether in playful activities or in the context of daily family routines. It is important that adoptive parents clearly understand the behaviors and attitudes that can be beneficial to children, and the socioemotional, relational, and developmental implications of taking on these same interactive behaviors that are more emotionally available.

Early parenting interventions, especially focusing on sensitivity (which is one of the dimensions of the EA), can be useful since they have been shown to be clinically effective in promoting secure attachment in children under 13 years (see the systematic review and meta-analysis of Wright and Edginton [94]). The evaluation of the effectiveness of interventions in disorganized attachment suggests that sensitivity-focused training was more effective than interventions providing representations, support focus, or a combination of the two (see the meta-analysis of Facompré et al. [95]). These types of programs can be an important resource for intervention. However, interventions that include EA could be useful since they integrate other important relational aspects. The basic principles of EA-based intervention are attachment, EA, and mindfulness [96]. AE-based intervention helps family members and other caregivers to learn about EA through psychoeducation, practicing mindfulness, examining their own attachment patterns, and reflecting on their dyadic interactions [9]. One asset of this program is that it is brief, simple (i.e., 4 to 6 sessions), profound, and can be implemented individually and/or in groups (see [9,66,97]).

As Clark et al. [7] have suggested, considering both EA and attachment measures in research are essential for developing preventive interventions that efficiently promote healthy family relationships. Attachment-based interventions promoting EA-based parenting skills have already demonstrated their effectiveness. The “Emotional Attachment and Emotional Availability (EA2) Intervention”, tele-intervention version [13], could be an important resource since it has been shown to be effective in increasing the responsiveness and involvement of the child in relation to adoptive parents (see [66]). The “Attachment-based Parenting Intervention VIPP-FC/A” also proved to be effective in promoting the emotional availability of girls and boys at early ages (i.e., *M* = 43.15 months), and in children who were previously institutionalized in relation to their adoptive mothers, increasing their involvement and responsiveness (see [66]). It may also be useful in older children and in adopted families.

## 5. Conclusions

To conclude, only a few studies have in recent times examined the association between EA and children´s secure attachment representations in adoptive families. The results of the present study, which was conducted in Portugal, contribute to the understanding of the factors that promote the adjustment of adoptive families, specifically the effects of the quality of the EA in adoptive parent-child interactive dyads, on the child´s secure attachment representations. Our findings provide a European perspective and contribute to the growing body of research showing that adoption can be a remarkably positive life event (see also [98]). The present study allows the identification of specific potential intervention aspects that promote attachment security in the new parental-child relationships that are established in adoptive families. The findings of this study suggest that EA (EA-parent and EA-child) can be a useful tool to construct and promote children´s secure attachment representations in adoptive families. It can also help policymakers, practitioners, and researchers to understand and to improve relational quality when responding to family needs in natural adoptive family contexts.

## Figures and Tables

**Table 1:** Descriptive statistics of children characteristics.

	*M*	*SD*	α
CASR	4.50	1.42	0.82
Children age ^a^	74.77	19.61	-
Age when adopted ^a^	21.85	17.60	-
Time elapsed since adoption ^a^	52.50	23.81	-

Notes. N = 26; CASR = child secure representations of attachment; ^a^ = estimated in months; *M* = mean; *SD* = standard deviation; α = Cronbach’s alpha.

**Table 2:** Descriptive statistics of parents’ emotional availability.

	Mother				Father				Parents			
	N	*M*	*SD*	α	N	*M*	*SD*	α	N	*M*	*SD*	α
EA-parent	26	22.09	3.14	0.82	23	21.38	3.61	0.83	49	21.76	3.35	0.82
Sensitivity	26	20.21	4.62	0.86	23	20.17	4.46	0.87	49	20.19	4.50	0.86
Structuring	26	19.69	4.73	0.79	23	18.39	5.75	0.89	49	19.08	5.22	0.85
Non-Intrusiveness	26	22.73	2.93	0.77	23	21.04	3.80	0.74	49	21.94	3.44	0.76
Non-Hostility	26	25.73	2.86	0.60	23	25.91	3.38	0.72	49	25.82	3.09	0.66
EA-child	26	21.63	3.08	0.85	23	19.46	3.81	0.88	49	20.61	3.57	0.88
Involvement	26	21.27 ^a^	3.16	0.70	23	18.91 ^a^	3.62	0.82	49	20.16	3.55	0.74
Responsiveness	26	21.98	3.42	0.70	23	20.00	4.40	0.75	49	21.05	4.00	0.77

Notes. EA = emotional availibility; *M* = mean; *SD* = standard deviation; α = Cronbach’s alpha. ^a^ = means differs at *p* < 0.05 (Mann-Whitney test).

**Table 3:** Correlations between secure representations of attachment and emotional availability for mothers (upper right corner) and fathers (lower left corner).

	1	2	3	4	5	6	7	8	9
1. CSRA	-	0.26	0.29	0.08	0.27	0.25	0.47 *	0.44 *	0.45 *
2. EA-parent	0.28	-	0.95 **	0.76 **	0.72 **	0.84 **	0.52 **	0.36	0.60 **
3. Sensitivity	0.36	0.97 **	-	0.64 **	0.68 **	0.82 **	0.61 **	0.45 *	0.68 **
4. Structuring	0.11	0.80 **	0.77 **	-	0.24	0.42 *	0.34	0.21	0.42 *
5. Non-Intrusiveness	0.18	0.73 **	0.66 **	0.28	-	0.64 **	0.32	0.22	0.38
6. Non-Hostility	0.31	0.79 **	0.76 **	0.37	0.67 **	-	0.40 *	0.27	0.46 *
7. EA-child	0.36	0.75 **	0.79 **	0.61 **	0.37	0.72 **	-	0.92 **	0.94 **
8. Involvement	0.46 *	0.67 **	0.72 **	0.48 *	0.34	0.71 **	0.94 **	-	0.74 **
9. Responsiveness	0.24	0.75 **	0.77 **	0.66 **	0.36	0.67 **	0.96 **	0.80 **	-

Notes. Correlations for mothers are above the diagonal; correlations for fathers are below the diagonal; CASR = child secure representations of attachment; EA = emotional availability; N = 26 for mothers; N = 23 for fathers. * *p* < 0.05; ** *p* < 0.01.

**Table 4:** Regression analyses.

	B	SE	*Beta*	*t*	*p*
Model 1					
	*F*(3,45) = 14.56; *p* < 0.001; R = 0.70; R^2^ = 0.49				
Intercept	−0.715	1.104		−0.648	0.520
EA-parent	0.094	0.045	0.221	2.061	0.045
Age when adopted ^a^	−0.022	0.009	−0.284	−2.62	0.012
Child age ^a^	0.048	0.008	0.638	5.873	0.000
Model 2					
	*F*(3,45) = 14.62; *p* < 0.001; R = 0.70; R^2^ = 0.49				
Intercept	−0.699	1.103		−0.634	0.530
EA-parent	0.093	0.045	0.219	2.048	0.046
Time elapsed since adoption ^a^	0.023	0.009	0.374	2.640	0.011
Child age ^a^	0.026	0.011	0.339	2.372	0.022
Model 3					
	*F*(3,45) = 3.53; *p* = 0.022; R = 0.43; R^2^ = 0.19				
Intercept	0.717	1.491		0.481	0.633
EA-child	0.172	0.056	0.432	3.063	0.004
Age when adopted ^a^	−0.010	0.011	−0.130	−0.965	0.339
Parents’ gender	0.298	0.398	0.106	0.748	0.458
Model 4					
	*F*(3,45) = 11.06; *p* < 0.0001; R = 0.61; R^2^ = 0.42				
Intercept	0.381	1.227		0.311	0.757
EA-child	0.105	0.05	0.265	2.117	0.040
Time elapsed since adoption ^a^	0.032	0.007	0.527	4.425	0.000
Parents’ gender	0.138	0.337	0.049	0.41	0.684

Notes. Dependent variable = secure representations of attachment; N = 49; E.A. = Emotional Availability; Gender: 1 = female, 2 = male; ^a^ = estimated in months; *B* = unstandardized beta; SE = standard error for the unstandardized beta; Beta = standardized beta; *t = t*-test statistic, *p* = probability value; R = multiple correlation coefficient; R^2^ = coefficient of determination; *F* = *F* statistic.

## Data Availability

Data are available for consultation when requested from the corresponding author, and with permission of the participants of the study.
